# REPRESENTATION LEARNING OF PROTEOME DYNAMICS TO CHARACTERIZE ORGANISMIC COMMUNICATION AND INTRINSIC HEALTH

**DOI:** 10.1093/geroni/igad104.0116

**Published:** 2023-12-21

**Authors:** Molei Liu, Bowen Xu, Sewanou Honfo, Alan Cohen, Martin Picard

**Affiliations:** Columbia Mailman School of Public Health, New York City, New York, United States; New York University, New York City, New York, United States; Universitéde Shebrooke, Sherbrooke, Quebec, Canada; Columbia University, New York City, New York, United States; Columbia University Irving Medical Center, New York City, New York, United States

## Abstract

Co-regulation and interactions among organ systems are crucial to maintain health. Therefore, understanding patterns of such organismic communication using proteome dynamics could be an innovative and effective strategy for quantifying intrinsic human health. In this preliminary study, we conducted a case-control study involving six participants with severe genetic mitochondrial disease and six age- and sex-matched healthy subjects. We measured and recorded the salivary proteome of each participant (n=2922 proteins) at 0-, 30-, and 45-minutes post-awakening as well as bedtime (pm), over two days, representing a natural physiological challenge known to involve systems-wide physiological recalibrations. We then analyzed the changes in protein levels across successive time points using a high-dimensional two-sample testing approach (Cai, Liu, and Xia 2013). The protein co-regulation pattern (captured by the covariance matrix of their changes in expression) was significantly different between the healthy and mitochondrial disease participants (p=0.01), thereby linking proteome dynamics with health status. We then implemented Weighted Correlation Network Analysis to identify representative features showing a significant correlation with the subject’s health status between pairs of successive time points (0-30, 30-45, 45-pm). The correlation between the extracted feature and the self-rated health was as high as 0.39 (p-value=0.03) for the 30-45 time period. This finding reveals an association between health status and salivary protein co-regulation patterns, and suggests a promising representation learning strategy to quantify the manifestation of health into accessible proteome dynamics.

